# More symptoms, less crime: distinguishing clinical severity from violence risk in a national follow-up study of adolescent forensic inpatients

**DOI:** 10.1186/s13034-026-01135-z

**Published:** 2026-07-16

**Authors:** Alfred Sarno, Riittakerttu Kaltiala

**Affiliations:** Faculty of Medicine and Health Technology, University of Tampere, Tampere, Finland

**Keywords:** Forensic psychiatry, Child and adolescent psychiatry, Recidivism, Violence, SAVRY, Risk assessment

## Abstract

**Background:**

The predictive value of symptom severity on post-discharge criminal offending in forensic psychiatric settings remains unknown, potentially leading to prolonged institutionalization of stabilized patients under the rationale of public safety. Furthermore, outcomes of adolescent forensic psychiatric inpatient care are understudied.

**Aims:**

To explore if clinical symptom severity predicts subsequent criminal offending, to investigate the role of severe disorders in future offending, and to quantify offence rates among former adolescent forensic inpatients compared to the general population.

**Methods:**

This national register-based study followed 289 youths (under 18 years) consecutively admitted to a forensic psychiatric inpatient unit in Finland between 2003 and 2019 for up to 18 years. Offence rates were analyzed using Cox proportional hazards regression, comparing patients against 1,149 matched general population controls. Predictors included the Brief Psychiatric Rating Scale (BPRS; score range 18–126 points) and Structured Assessment of Violence Risk in Youth (SAVRY), both assigned during a two-month evaluation.

**Results:**

Former patients had a significantly higher hazard of offending than controls (HR 6.67, 95% CI 5.10–8.72). However, higher clinical symptom severity (BPRS) independently reduced the risk of future offending by 2% per point (HR = 0.98, 95% CI 0.96-1.00, *p* = .025); e.g., a 20-point difference corresponds to an approximate 34% reduction in risk. High-risk patients (SAVRY) with severe symptoms offended at rates similar to low-risk patients. Personality disorders were associated with subsequent offending (HR = 1.92, 95% CI 1.01–3.66, *p* = .047), while schizophrenia spectrum disorders and Length of Stay were not.

**Conclusions:**

Severe psychopathology predicts reduced subsequent offending after forensic adolescent inpatient care, possibly due to treatment responsiveness, systemic safety nets and functional impairment. Prolonging hospitalization under the rationale of public safety based on symptom severity may be unnecessary for stabilized patients if continuity of care and outpatient supervision are ensured.

**Supplementary Information:**

The online version contains supplementary material available at https://doi.org/10.1186/s13034-026-01135-z.

## Introduction

### Subsequent criminal offending after forensic psychiatric care or justice-involvement

In forensic psychiatry, the relationship between clinical pathology and public safety can be counterintuitive. While severe psychotic symptoms and dangerous behavior drive the initial admission to secure care, evidence suggests that acute psychopathology does not drive long-term criminal offending in these treated populations [1, 2]. In fact, patients with the most severe diagnostic profiles often demonstrate lower rates of reoffending compared to those with personality disorders or substance use needs [3–5]. Additionally, patients discharged from secure forensic inpatient units often demonstrate lower reoffending rates than released prisoners or other adjacent groups in both adult and adolescent populations. This pattern is widely attributed to specific features of forensic care, including patient selection, lengthy inpatient treatment, step-down placements, and legally enforceable outpatient conditions [6, 7].

Predicting which patients remain at risk after discharge, however, remains a challenge. Research primarily conducted on adult populations has identified a group of static demographic and criminological predictors that correlate with subsequent offending, such as younger age at discharge, male sex, and a history of prior convictions [8, 9]. Structured professional judgment tools, such as the Historical-Clinical-Risk Management-20 (HCR-20) for adults and the Structured Assessment of Violence Risk in Youth (SAVRY) for adolescents, have also demonstrated utility in predicting subsequent offending [10, 11]. Yet, when considering clinical variables, literature presents a seemingly contradictory picture regarding the risk posed by arguably forensic psychiatry’s central diagnosis: psychotic disorders, and the role of symptom severity.

In the general population and broad justice-involved cohorts, schizophrenia spectrum disorders are linked to increased participation in violent crime and recidivism, especially when compared to non-disordered counterparts [12–16]. However, within forensic psychiatric populations, this correlation is frequently absent or reversed [2]. When forensic patients with psychosis are compared to those with personality disorders (PD) or substance use disorders (SUD), the psychotic group often emerges as the lower-risk cohort. For instance, large-scale meta-analyses and longitudinal cohorts in Sweden, Denmark, and the UK have found that diagnoses of bipolar disorder, substance use, and particularly personality disorders confer significantly higher hazards of reoffending than schizophrenia spectrum disorders [1, 3, 17, 18]. Similarly, a study of prisoners released from a Medical Correction Center in Japan found that those with schizophrenia were consistently less likely to recidivate than prisoners with other disorders [5].

This inverse relationship in forensic settings, where psychosis appears protective compared to other diagnoses, may be explained by the responsiveness of the condition to treatment. Authors suggest that violence driven by acute psychotic symptoms responds favorably to medication and clinical management, whereas violence driven by antisocial personality traits or other criminogenic factors is more static and resistant to medical intervention [7, 19]. The forensic system also often imposes more rigorous outpatient supervision and medication monitoring for patients with psychotic disorders, thereby reducing the opportunity for recidivism compared to less intensively managed offender groups [5].

Beyond diagnosis, the predictive value of symptom severity remains unclear. In a justice-involved cohort of adults discharged from correctional facilities, Jones et al. [14] employed the Brief Psychiatric Rating Scale-Expanded Version (BPRS-E) during the index incarceration and found that higher symptom severity predicted higher rates of subsequent reincarceration upon return to the community. While high symptom loads in released prisoners may represent a risk, in forensic settings, acute psychopathology might conversely signal a need that can be effectively tended to through medical treatment, unlike antisocial traits for example.

Research specifically regarding adolescents in this context is especially scarce. In a general psychiatric inpatient setting, a large Finnish registry study parallels the adult findings, noting that adolescents with schizophrenia group diagnoses had the lowest risk for subsequent crime, while those with conduct, personality, and substance use disorders carried the greatest risk [4]. This suggests that for adolescents, as with adults, acute psychopathology may represent a treatable need rather than a fixed risk factor, particularly when discharge includes strict monitoring.

### Research gap

To our knowledge, no studies have been published specifically addressing (1) whether symptom severity as measured by clinical scales (such as the BPRS) is associated with subsequent offending in forensic psychiatric patients generally – a gap relevant to the field at large – or (2) the specific rates and predictors of subsequent offending following *adolescent* forensic inpatient psychiatric treatment. Thus, data regarding reoffending rates, crime typologies, and risk predictors in this specific population remains largely unknown. Compared to many adult forensic inpatient settings, which may be legally restricted to treating psychotic disorders, adolescent units are generally more diagnostically heterogeneous [20], therefore well-suited for between-diagnosis comparisons.

Clarifying these predictors is beneficial not only for public safety but for targeted and ethical management of forensic patients. If acute psychopathology is associated with lower long-term criminal offending, it could suggest that prolonging treatment beyond sufficient symptom remission may be unnecessary, also underscoring the importance of outpatient supervision and medication monitoring. Recognizing this distinction could guide minimizing infringement on personal liberties and optimizing the allocation of healthcare resources. Additionally, suggesting a potential for validating a routinely administered, accessible instrument like the BPRS as a predictive tool would offer immediate clinical utility for risk stratification.

### Purpose of our study

The present study aimed to bridge the gap in forensic literature through two primary objectives:

1. To determine the prevalence and typology of criminal offending (general and violent) in adolescents following discharge from adolescent forensic psychiatric inpatient treatment compared to general population controls.
2. To investigate whether symptom severity (measured by the BPRS) or a diagnosis of a severe disorder (schizophrenia spectrum) compared to other diagnoses associated with reduced offending, while contextualizing these clinical variables against an established criminogenic risk measure (the SAVRY).

## Method

### Setting

The study setting is the Adolescent Forensic Unit in Tampere University Hospital, Finland, one of two comparable nationally serving adolescent forensic psychiatric inpatient units in the country. The 12-bed unit serves to assess and treat minors who present with severe mental disorders complicated by dangerous and treatment-resistant violent behaviour. Analogous to adult forensic psychiatric services in Finland, the core functions of the unit are (1) formal, court-ordered forensic psychiatric assessments of minors who have committed severe criminal offences (usually violent) and are of age of criminal responsibility (15 years old), (2) court-ordered psychiatric care for adolescent offenders, and (3) civil psychiatric inpatient care for minors with severe mental disorders and associated violent behaviour, mainly on involuntary basis. All treatment is provided on an inpatient basis, continuing until the patient is clinically stabilized and deemed safe for transition to outpatient care and, typically, supported child welfare placements.

### Design

A register-based follow-up study of patients admitted to the adolescent forensic psychiatric inpatient unit since its inception in 2003 to 2019.

From the database of Tampere University Hospital, information accumulated during the index admission to the adolescent forensic unit was collected. This included dates of admission and discharge, sex, age at admission, psychiatric diagnoses recorded at discharge, symptom severity rating, and violence risk rating carried out by the multi-disciplinary clinical team during the two-month evaluation phase included in all admissions to the unit. This information is routinely accumulated to the research database used in this study. In case of missing data, the authors collected the information retrospectively from original clinical case files. In the rare case of forensic rehospitalization, only the first stay was included in the study.

For each patient, four general population controls matched for sex, birth year, and birthplace were identified from the Population Information System. Each control was assigned the index date of their corresponding patient. To analyze subsequent offending, data regarding the offence title, sentencing date, and sentence type were obtained from the Legal Register Centre. Dates of death for both patients and controls were retrieved from the Causes of Death Registry maintained by Statistics Finland.

### Study population

The study population is described in detail in Sarno & Kaltiala [20, 21] along with its demographic, psychosocial and psychiatric variables. During the inclusion period, 313 young people (< 18 years) were admitted to the study unit. Their median age at admission was 15.4 (IQR = 2.44) years. The most common diagnoses (primary and non-primary) belonged to the conduct disorder group (F90-99; 61.7%), schizophrenia spectrum group (F20-29; 31.3%) and developmental disorders group (F80-89; 26.5%). The majority had more than one F-diagnosis. An overwhelming majority were known to have committed violent and/or criminal acts (81.7%), and the majority had a history of significant suicidal behaviour (61.5%). The median length of stay was 133 days (IQR = 213) [20]. 

The final analysis cohort comprised 289 patients for whom the population information system returned 1,149 matched controls. The discrepancy between the total admissions and the analyzed cohort is due to registry-level exclusions, including a lack of suitable matched controls, research opt-outs, or changes in personal identity codes. When reporting statistical data, the threshold for k-anonymity value was set at 3.

### Crime categorization

Offense data obtained from the Legal Register Centre were recorded according to seven standard legislative categories: (1) offenses against property, (2) offences against life and health, (3) sexual offences, (4) offences against public authority and perjury, (5) traffic offences, (6) other offences against the Penal Code, and (7) offences against other Acts.

We also constructed three aggregate variables: any offence, violent offences and substance-related offences. The “any offence” category comprised all convictions across the seven categories listed above. The violent crime category comprised acts involving intentional physical harm or threat. This included robbery or extortion, all sexual offences, resistance to a public official, murder, manslaughter, infanticide, assault, aggravated assault or brawling, while excluding acts of negligence. The substance-related category combined driving while intoxicated with narcotics offences and violations of the Alcohol Act.

Entries with unknown offence classifications were excluded. Additionally, we removed all legal outcomes that did not constitute a finding of guilt, including acquittals, dismissed charges, discontinued proceedings, and cases ruled inadmissible or resolved without a substantive pronouncement. Finally, to ensure the dataset reflected clinically and socially significant offending, we excluded minor infractions punishable solely by a summary penal fee, such as minor traffic violations or littering, which do not constitute a criminal record entry in the same manner as court-imposed sentences. Consequently, the final dataset comprised only offences where a definitive criminal sentence or sanction was imposed.

### Measures

#### Brief Psychiatric Rating Scale (BPRS-18)

The primary predictor variable was the Brief Psychiatric Rating Scale (BPRS-18), an instrument widely utilized for the standardized documentation of a broad range of psychiatric symptoms (total score range 18–126 points) [22].

BPRS scores were completed at the end of the two-month assessment period. Although the BPRS is designed as a dynamic state measure, potentially limiting its utility for long-term prediction, its application in this study likely captures more enduring psychopathology. This is because scores were finalized only after a comprehensive two-month assessment period, often following extensive prior treatment, therefore reflecting more of a residual symptom load rather than acute, transient distress.

Around 5% of the BPRS values were missing at random and were filled in using single imputation method [23].

#### Structured assessment of violence risk in youth

To contextualize the predictive value of clinical symptom severity (BPRS), we employed the Structured Assessment of Violence Risk in Youth (SAVRY) as a secondary predictor and established benchmark [10].

The SAVRY is a Structured Professional Judgment (SPJ) tool that captures established criminogenic risk factors and is widely validated for use with adolescents [10]. It evaluates 24 risk items across historical, social/contextual, and individual domains, as well as protective factors. A total risk score (range 0–48) was calculated by summing the individual risk items [10].

SAVRY scores were available for approximately 85% of the cohort; missing data were retrospectively scored by the authors using comprehensive multidisciplinary medical records prior to having access to offending data [20].

#### Covariates

In addition to the primary and secondary predictors, data were collected on variables known to predict recidivism in numerous settings [2, 24]. These variables included sex, age at admission, primary diagnosis (ICD-10 F-codes assigned at discharge, separated into groups for analysis) and length of the stay (measured in days) and a pre-index criminal history, which was defined as any criminal sanction received prior to or during the index year.

Notably, Substance Use Disorders (SUD) were not explicitly modeled as a diagnostic covariate. Within this highly impaired forensic cohort, SUD was exceptionally rare as a *primary* diagnosis, falling below the registry’s statistical reporting threshold. Clinically, this aligns with the severe functional impairment and prolonged institutionalization characteristic of this group, which heavily restricts their capacity and opportunity to procure illicit substances compared to standard justice-involved youth.

#### Analysis

Data were processed using IBM SPSS v26.

All statistical analyses were evaluated for significance using a two-tailed alpha level of 0.05.

The follow-up period started after the patient was admitted to the Adolescent Forensic Unit (index date) and lasted either until death or 30.03.2023.

Cox proportional hazards regression analyzed the time to subsequent offending. The outcome variable was defined by the year of sentencing, as exact dates of offence commission were not available in the registry. Thus, the time scale was measured in years to align with the data precision. To strictly assess post-index offending, sentences issued prior to or during the index year were classified as criminal history, while only those issued after the index year were counted as subsequent offending events. A separate procedure was not performed for violent offending due to the substantial overlap between the two outcomes, as violent offending constituted the overwhelming majority of general offending cases (67%).

To compare the crime rate of the patient cohort with the controls, a Hazard Ratio (HR) was calculated. To account for varying reporting in other studies, incidence rates were also calculated per 100 person-years.

Primary and secondary predictors, along with the covariates were entered in separate univariable Cox regression models. Following the univariable analyses, predictors demonstrating a statistically significant association with subsequent offending were included in a subsequent multivariable Cox regression model to adjust for confounding. The individual SAVRY risk domains were excluded from the multivariable analysis to avoid multicollinearity. Results from the Cox regression are presented as a Hazard Ratio (HR) with a 95% CI.

The proportional hazards assumption was assessed prior to interpreting the multivariable Cox regression model. For categorical covariates, the assumption was evaluated through visual inspection of log-minus-log survival curves. For the continuous predictor (BPRS), the assumption was tested by creating a time-dependent covariate, defined as the interaction between the predictor and survival time, and including it in the model.

To supplement the analysis and visually investigate the interaction between criminogenic risk and clinical symptom severity, we stratified the cohort into four subgroups based on median splits of the SAVRY Total Score and BPRS Total Score. Patients were assigned to “High” or “Low” categories based on their position relative to these sample-specific medians rather than predefined clinical cut-offs. Survival distributions for these four profiles were estimated using the Kaplan-Meier method and compared using the Log Rank (Mantel-Cox) test. Post-hoc pairwise comparisons were conducted to identify specific differences between the strata.

## Results

### Sample and offending statistics

Matched general population controls were returned for 289 patients (92.3%) and their 1149 controls. The follow-up period ranged from 1 to 18 years (median 8 years; IQR = 9). During the follow-up period, 19 patients (6.1%) had died, compared to 5 deaths (0.4%) among the controls, affecting the period’s length.

As detailed in Table 1, the patient cohort exhibited a significantly higher prevalence of criminal behaviour compared to general population controls. Prior or during the index year, 22.1%^1^ of patients had a recorded criminal history, compared to only 1.0% of controls (*p* <.001). During the follow-up period, this disparity persisted, with 42.6% of former patients committing at least one new offence compared to 8.4% of controls (*p* <.001).

**Table 1:** Prevalence of criminal offending among patients and general population controls with statistical comparisons

Variables	Patients (*N* = 289) *n* (% Total) [% Off.]	Controls (*N* = 1149) *n* (% Total) [% Off.]	Comparison (*p*) Prev. [Prof.]
**Pre-Index Offending**			
Any crime (pre-index)	64 (22.1) [–]	12 (1.0) [–]	< 0.001 [–]
**Post-Index Offending**			
Any crime (post-index)	123 (42.6) [100.0]	97 (8.4) [100.0]	< 0.001 [–]
*Specific Offence Types*			
Offences against property	87 (30.1) [70.7]	27 (2.4) [27.8]	< 0.001 [< 0.001]
Offences against life and health	73 (25.3) [59.3]	20 (1.7) [20.6]	< 0.001 [< 0.001]
Sexual offences	10 (3.5) [8.1]	5 (0.4) [5.2]	< 0.001 [0.549]
Offences against public auth./perjury	43 (14.9) [35.0]	7 (0.6) [7.2]	< 0.001 [< 0.001]
Traffic offences	42 (14.5) [34.1]	58 (5.0) [59.8]	< 0.001 [< 0.001]
Other offences against Penal Code	84 (29.1) [68.3]	27 (2.4) [27.8]	< 0.001 [< 0.001]
Offences against other Acts	26 (9.0) [21.1]	11 (1.0) [11.3]	< 0.001 [0.081]
*Aggregated Categories*			
Violent crimes	83 (28.7) [67.5]	26 (2.3) [26.8]	< 0.001 [< 0.001]
Substance-related offences	70 (24.2) [56.9]	49 (4.3) [50.5]	< 0.001 [0.419]

% Total, Prevalence within the total group (*N*)

[% Off.], Proportion of offenders (those who committed *Any crime* post-index) within that group who committed the specific offence type

Prev., P-value for difference in Total prevalence

[Prof.], P-value for difference in Offender Profile. *p*-values are derived from Chi-square or Fisher’s exact tests

Subsequent offending risk was substantially higher for the patient cohort (HR = 6.67, 95% CI 5.10–8.72).

The patient cohort accumulated 1,783 person-years of follow-up, during which the incidence of any criminal offending was 6.9 per 100 person-years, while the control group accumulated 10,281 person-years, yielding an incidence rate of 0.94 per 100 person-years.

Among those who offended, patients were significantly more likely to commit severe offences, including violent crimes (67.5% of patient offenders vs. 26.8% of control offenders, *p* <.001). Conversely, while traffic offences were more prevalent in the patient group overall (14.5% vs. 5.0%), they constituted the majority of crimes committed by the control group offenders (59.8%), whereas they represented a significantly smaller proportion of the offending profile among patient offenders (34.1%, *p* <.001).

The patient cohort offended more rapidly than the controls. The median time to the first post-index offence was significantly shorter for patients (3.0 years, IQR = 4.0) compared to controls (4.0 years, IQR = 3.0; *p* <.001).

### Unadjusted predictors of subsequent offending among former patients

The full results of the univariable Cox regression analyses are presented in Supplemental Table S1.

Regarding the primary and secondary predictors, a higher BPRS Total Score was associated with a reduced risk of subsequent offending (HR = 0.97, *p* <.001. Conversely, the SAVRY Total Score predicted a higher risk of subsequent offending (HR = 1.06, *p* <.001), with the Individual/Clinical domain being the strongest driver of this risk (HR = 1.20, *p* <.001).

In terms of diagnoses, schizophrenia spectrum disorders (F20–29) were protective (HR = 0.40, *p* <.001), whereas personality disorders (F60–69; HR = 1.90, *p* =.025) and behavioural disorders (F90–98; HR = 1.99, *p* <.001) increased the risk of future offending.

Among other covariates, male sex (HR = 2.35, *p* <.001) and criminal history (*n* = 64; HR = 2.71, *p* <.001) were risk factors for subsequent offending. Similarly, a longer Length of Stay was protective (in days; HR = 0.999, *p* =.007).

### Independent predictors of subsequent offending among former patients

The full results of the multivariable Cox regression analyses are presented in Table 2.

**Table 2:** Multivariable cox proportional hazards regression model predicting general subsequent offending (*N* = 289)

Predictor	*n* (%)	HR	95% CI	*p*
**Demographic and Background**				
Male sex	–	1.37	[0.88, 2.15]	0.164
Criminal history (pre-index)	64 (22.1)	2.15	[1.43, 3.23]	< 0.001
**Clinical Characteristics**				
Length of stay (days)	–	1.00	[0.99, 1.00]	0.629
BPRS Total Score	–	0.98	[0.96, 1.00]	0.025
**Psychiatric Diagnoses**				
Schizophrenia spectrum (F20–29)	75 (26.0)	0.84	[0.44, 1.61]	0.606
Personality disorders (F60–69)	24 (8.3)	1.92	[1.01, 3.66]	0.047
Behavioural/onset childhood (F90–98)	100 (34.6)	1.52	[0.97, 2.37]	0.068
**Risk Assessment**				
SAVRY Total Score	–	1.05	[1.03, 1.08]	< 0.001

HR, Hazard Ratio; CI, Confidence Interval; BPRS, Brief Psychiatric Rating Scale; SAVRY, Structured Assessment of Violence Risk in Youth

Multicollinearity was assessed for all predictors in the model: no issues were detected, with all Variance Inflation Factors (VIF) falling well below the threshold of 5 (Range: 1.06–1.86)

**Symptom severity** remained a protective factor; a higher BPRS Total Score was associated with a lower risk of subsequent offending (HR = 0.98, *p* =.025). The SAVRY Total Score also retained its predictive validity (HR = 1.05, *p* <.001).

Among the diagnostic categories, **Personality disorders (F60–69)** remained associated with future offending (HR = 1.92, *p* =.047). **Criminal History** also remained a strong predictor, with prior offending more than doubling the hazard of subsequent offending (HR = 2.15, *p* <.001).

However, the significant positive effects of Male sex and Behavioural disorders (F90–98), and the inverse effect of Schizophrenia spectrum disorders (F20–29), disappeared in the multivariable model.

The model was checked for multicollinearity, and all Variance Inflation Factors (VIF) were low (< 2.0), indicating no issues with intercorrelations among predictors.

The proportional hazards assumption was met for all covariates. Visual inspection of the log-minus-log plots for categorical variables demonstrated approximately parallel curves, indicating no violation of proportionality. Furthermore, the time-dependent interaction term for the primary continuous predictor, symptom severity (BPRS), was not statistically significant (*p* =.461), indicating that the hazard ratio remained stable over the follow-up period.

### Stratified survival analysis

**Fig. 1:**
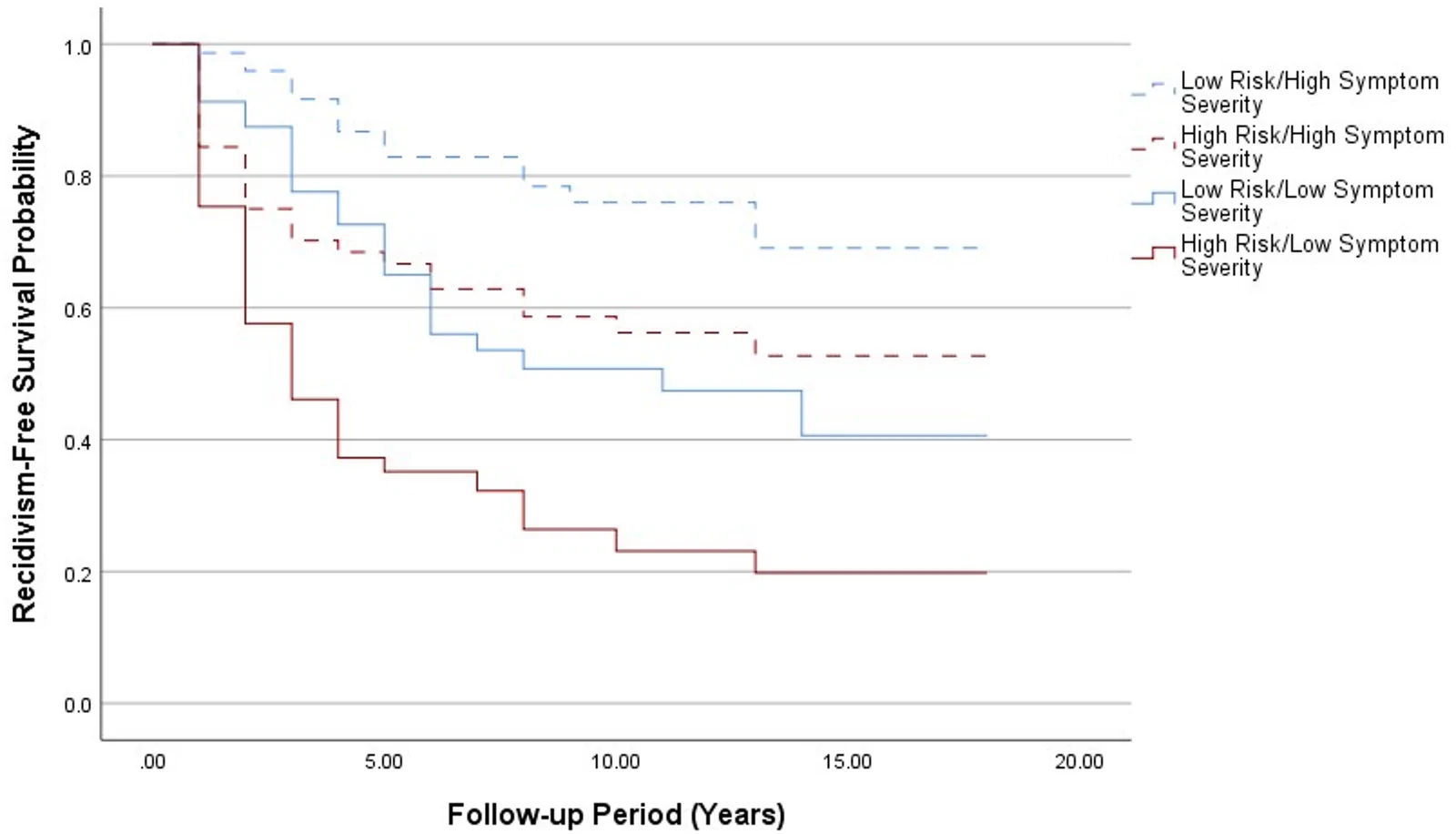
Kaplan-Meier Survival Estimates for Subsequent Offending by Risk (SAVRY) and Symptom (BPRS) Profile (*n* = 287). The plot illustrates time to first subsequent offending event for four subgroups defined by median splits of the SAVRY and BPRS total scores. The **High Risk/Low Symptom Severity** group (Solid Red Line; *n* = 69) showed the lowest survival probability. The **Low Risk/High Symptom Severity** group (Dashed Blue Line; *n* = 74) showed the highest survival probability. Pairwise comparisons confirmed that the **High Risk/High Symptom Severity** group (Dashed Red Line; *n* = 64) was not significantly different from the **Low Risk/Low Symptom Severity** group (Solid Blue Line; *n* = 80; *p* =.737). “High” and “Low” designations are relative to the cohort’s median values for research purposes and do not represent absolute clinical risk thresholds or standardized diagnostic categories. Two patients were excluded from this stratified survival analysis due to missing SAVRY or BPRS data that could not be retrospectively scored (e.g., due to premature discharge)

The Kaplan-Meier analysis revealed differences in offence-free survival rates across the four risk-symptom profiles (χ2 = 48.06, df = 3, *p* <.001; Fig. 1). Post-hoc pairwise comparisons are presented in Supplemental Table S2.

The High Risk/Low Symptom Severity (HR/LSS; High SAVRY/Low BPRS) group (*n* = 69) demonstrated the most rapid rate of offending, differing significantly from all other groups (all *p* <.001). This profile represented the worst-case scenario for future offending, with approximately 68% of the group offending during the follow-up period.

Conversely, the LR/HSS group (*n* = 74) exhibited the highest rate of survival, with only 20% of patients offending. This group was significantly “safer” than even the baseline LR/LSS group (*n* = 80; *p* =.002), suggesting that high symptom severity exerts a protective effect beyond the mere absence of criminogenic risk factors.

Notably, the presence of high symptom severity appeared to minimize the risk associated with high SAVRY scores. The HR/HSS group (*n* = 64) showed a survival trajectory that was statistically indistinguishable from the LR/LSS group (*p* =.737).

## Discussion

### Main findings

To our knowledge, this is the first study to examine subsequent offending rates among adolescent forensic psychiatric inpatients and the first study in any forensic psychiatric population (adult or adolescent) to investigate the predictive utility of a standardized clinical symptom scale (BPRS) for future offending.

**Former adolescent forensic inpatients carried a higher risk of subsequent offending (especially violent) than matched general population controls**. However, offending rates were lower than those typically reported for general justice-involved adolescents [12, 25, 26]. For example, a recent Australian study by Akpanekpo and Butler [12] found that 60.9% of justice-involved adolescents reoffended generally, and 37.0% reoffended violently, within a short 24-month follow-up.

In regards to non-forensic psychiatric adolescent inpatient populations, Kaltiala et al. [4] reported an overall crime incidence of 2.4 per 100 person-years for adolescents discharged from civil psychiatric care in Finland. Our rate of 6.9 is nearly three times higher, confirming that adolescent forensic patients represent a high-severity segment of the psychiatric population. However, our rate remains notably lower than the specific “high-risk” subgroups identified in the non-forensic setting, such as those with conduct or substance use disorders (F90-92, F10-19), who exhibited incidence rates of 11.5 per 100 person-years. Thus, while forensic patients are elevated in risk compared to the “average” psychiatric adolescent inpatient, they appear to reoffend less frequently than the externalizing profiles treated in civil settings.

**Schizophrenia Spectrum Disorders were not an independent predictor of reoffending**. This finding diverges from studies in justice-involved or general populations, where psychosis is often identified as a risk factor, especially when compared to non-disordered counterparts [12–16]. However, it aligns with findings in adult forensic psychiatry and general adolescent psychiatric inpatient samples [1, 3, 4, 7, 17, 18]. This likely reflects treatment responsiveness (psychotic symptoms are often amenable to medication), outpatient supervision and medication monitoring imposable by the forensic system [4–6]. In our cohort, patients with personality disorders carried nearly double the risk of subsequent offending, as the criminogenic mechanisms in these disorders, such as impulsivity and antisocial attitudes, are often more static and resistant to pharmacotherapy [19].

The BPRS demonstrated significant predictive validity, indicating that **higher levels of general psychopathology predicted a lower risk of subsequent offending**. While a Hazard Ratio of 0.98 represents a 2% reduction in risk per point, this accumulates to a clinically substantial reduction in risk across the broad range of symptom severity typical of this population (e.g., a 20-point difference corresponds to an approximate 34% reduction in risk). High-risk patients with severe symptoms offended at rates similar to low-risk patients (Fig. 1). This protective effect is possibly mediated by (1) treatment responsiveness, (2) the systemic response to illness, and (3) functional impairment.

First, a high BPRS score can reflect violence driven by acute psychopathology which is often more reactive rather than proactive in nature [27]. As with the treatment responsiveness observed in our psychosis findings, when the underlying illness is treated, the driver for aggression subsides. In contrast, patients with lower symptom loads (often those with personality or conduct disorders) may engage in violence driven by static, instrumental antisocial traits which persist even when the patient is clinically stable.

Second, the systemic response to evident illness creates a safety net; patients with more severe psychopathology likely signal a need for more intensive medical treatment, often enforceable outpatient monitoring and lower thresholds for readmission compared to those who appear clinically well but harbor criminogenic traits. Additionally, patients with high symptom loads are less often able to live independently; instead, they are typically transferred to supported housing units or step-down facilities that provide supervision, rehabilitation, and daily support. This structural scaffolding in part physically limits the risk of offending.

Third, the protective effect of high symptom scores possibly reflects functional impairment [28], as the severe psychiatric symptoms captured by elevated BPRS scores, such as conceptual disorganization, emotional withdrawal, or blunted affect, may functionally limit the executive capacity required to plan and execute criminal acts [28, 29].

### Other clinical and demographic predictors

Initially in line with extensive forensic literature [2, 24], male sex was a strong predictor of recidivism in our unadjusted analyses. However, this effect lost statistical significance in the multivariable model (*p* =.164). This suggests that the risk typically attributed to male gender may be mediated by other variables captured in our model, such as higher risk scores on the SAVRY among males. Similarly, while younger age is consistently identified as a robust risk factor for recidivism in adult forensic cohorts [24] and general offender populations [2], it did not emerge as a significant predictor in our study. This likely reflects the fact that younger adolescents in our cohort are cared for by social services for longer a duration, subject to supervision often until the age of majority. In addition to providing physical limits to offending, this allows younger patients to undergo neurobiological maturation within a stabilizing environment, capitalizing on a developmental potential that is somewhat more limited in older adolescents [30]. Thus, this extended period of institutional oversight may mitigate the risk traditionally associated with early-onset offending in this group.

Finally, regarding Length of Stay, while our unadjusted analysis suggested a protective effect for longer hospitalization, this association disappeared in the multivariable model, contrasting previous literature [2, 24]. This is possibly because Length of Stay functions as a proxy for symptom severity, the primary driver of treatment duration in this cohort [20]. Thus, extending hospitalization for adolescents does not appear to independently mitigate the long-term risk of subsequent offending once other risk factors are accounted for.

### Strengths and limitations

Key strengths include a comprehensive national cohort covering roughly half of Finland’s adolescent forensic patients, a considerable follow-up time and high-quality registers that minimized attrition.

Limitations involve retrospective scoring of some SAVRY data, which may underrepresent protective factors. A fraction of BPRS values were missing and handled via single imputation which may not capture the clinical nuance of missing data. Furthermore, while the overall sample size was sufficient to detect the primary association between continuous symptom severity and offending, the multivariable model may have been underpowered to detect independent effects within smaller subgroups. Consequently, the wide confidence intervals observed for personality disorders, as well as the loss of statistical significance for sex and behavioural/onset childhood disorders in the adjusted model, may partially reflect sample size constraints. Procedurally, the age of criminal responsibility (15 years) likely underestimates pre-index offending, and reliance on sentencing years rather than offense dates may have misclassified some early recidivism as prior history. Additionally, the BPRS was originally developed and validated for adult populations. While utilized in clinical practice for adolescents, its psychometric properties in youth are less established.

Finally, while this national cohort is diagnostically heterogeneous, encompassing both civilly committed and adjudicated youth, as well as individuals evaluated but not retained for long-term care, Finland’s system predominantly targets severe mental disorders (e.g. early-onset schizophrenia, which typically represents a particularly severe and treatment-resistant phenotype [31]**)** and utilizes strong outpatient monitoring. Thus, these findings may manifest differently in international jurisdictions with less structured step-down aftercare, or in juvenile forensic populations dominated by conduct or substance use disorders. However, because admission to Finnish Adolescent Forensic units is based primarily on clinical need rather than legal thresholds, the cohort captures a broad spectrum of adolescent psychiatric morbidity, preserving its relevance to diverse international contexts.

## Conclusion

This study provides novel longitudinal evidence regarding the outcomes of adolescent forensic psychiatric care, revealing that severe clinical symptoms predict **reduced** subsequent offending, possibly due to the responsiveness of these symptoms to treatment, the robust systemic safety nets triggered by severe psychopathology and the functional impairment associated with severe illness.

Clinical practice and legislation warrants a clear distinction between treatment needs and public safety risks. Optimizing outcomes requires avoiding prolonged hospitalization for stabilized patients with high symptom loads, provided continuity of care is ensured, while intensifying long-term community supervision and behavioural interventions for the lower-symptom, high-risk subgroup. By distinguishing psychopathology from risk, forensic services can more effectively align resource allocation with the actual drivers of future offending.

## Supplementary Information

Supplementary Material 1.

## Data Availability

Data for the current study are the property of Statistics Finland, Finnish Social and Health Data Permit Authority Findata and the Legal Register Centre of Finland. The data are available from these authorities, but restrictions apply.

## Footnotes

1 Although 81. % of the cohort had a history of criminal behavior, only 22. % had a registered criminal history. This discrepancy stems from the Finnish age of criminal responsibility of 15 years (median age at admission was 15.4) precluding sanctions for earlier acts, as well as the fact that not all known criminal activity was previously reported to authorities.
