# Supplementary material for: More symptoms, less crime: distinguishing clinical severity from violence risk in a national follow-up study of adolescent forensic inpatients
Source: Child Adolesc Psychiatry Ment Health. 2026 Jul 16;20:141. doi: 10.1186/s13034-026-01135-z (PMC13670630; doi:10.1186/s13034-026-01135-z)
Supplement: Supplementary file 1 [file 13034_2026_1135_MOESM1_ESM.docx]

**Table S1**

*Univariable Cox Proportional Hazards Regression Models Predicting Subsequent Offending (N = 289)*

| Predictor | *n* (%) | *HR* | 95% CI | *p* |
| --- | --- | --- | --- | --- |
| **Demographic & Background** |  |  |  |  |
| Male sex | – | 2.35 | [1.55, 3.57] | < .001 |
| Age at admission (years) | – | 0.96 | [0.88, 1.05] | .383 |
| Criminal history (pre-index) | 64 (22.1) | 2.71 | [1.85, 3.96] | < .001 |
| **Clinical Characteristics** |  |  |  |  |
| Length of stay (per day) | – | 1.00 | [0.998, 1.00] | .007 |
| BPRS Total Score (per point) | – | 0.97 | [0.96, 0.98] | < .001 |
| **Psychiatric Diagnoses (ICD-10)** |  |  |  |  |
| Schizophrenia spectrum (F20–29) | 75 (26.0) | 0.40 | [0.24, 0.65] | < .001 |
| Mood disorders (F30–39) | 21 (7.3) | 0.93 | [0.47, 1.83] | .832 |
| Neurotic/stress-related (F40–48) | 7 (2.4) | 0.05 | [0.00, 9.60] | .261 |
| Physiological/physical (F50–59) | 8 (2.8) | 0.57 | [0.14, 2.31] | .432 |
| Personality disorders (F60–69) | 24 (8.3) | 1.90 | [1.08, 3.32] | .025 |
| Intellectual disabilities (F70–79) | 8 (2.8) | 1.78 | [0.73, 4.37] | .205 |
| Psychological development (F80–89) | 35 (12.1) | 0.81 | [0.45, 1.47] | .488 |
| Behavioral/onset childhood (F90–98) | 100 (34.6) | 1.99 | [1.39, 2.83] | < .001 |
| **Risk Assessment (SAVRY)** |  |  |  |  |
| SAVRY Total Score (per point) | – | 1.06 | [1.03, 1.08] | < .001 |
| Historical Domain (per point) | – | 1.09 | [1.05, 1.14] | < .001 |
| Social/Contextual Domain (per point) | – | 1.06 | [0.99, 1.14] | .114 |
| Individual/Clinical Domain (per point) | – | 1.20 | [1.13, 1.27] | < .001 |
| Protective Domain (per point) | – | 0.99 | [0.88, 1.10] | .825 |

*Note.* HR = Hazard Ratio; CI = Confidence Interval; BPRS = Brief Psychiatric Rating Scale; SAVRY = Structured Assessment of Violence Risk in Youth. HR for continuous variables represents the change in risk for every 1-unit increase. Substance use disorders (F10–19) were excluded from reporting due to low case counts.

**Table S2**

*Pairwise Comparisons of Survival Distributions (Log Rank Test) for the Four Risk-Symptom Profiles (N = 288)*

| Profile Group (n) | Comparison Group | Chi-Square | df | Sig. (p) |
| --- | --- | --- | --- | --- |
| **High Risk / Low Symptom**  (69) | Low Risk / High Symptom | 42.295 | 1 | < .001 |
|  | High Risk / High Symptom | 12.759 | 1 | < .001 |
|  | Low Risk / Low Symptom | 14.358 | 1 | < .001 |
| **Low Risk / High Symptom**  (74) | High Risk / Low Symptom | 42.295 | 1 | < .001 |
|  | High Risk / High Symptom | 6.737 | 1 | .009 |
|  | Low Risk / Low Symptom | 9.679 | 1 | .002 |
| **High Risk / High Symptom**  (64) | High Risk / Low Symptom | 12.759 | 1 | < .001 |
|  | Low Risk / High Symptom | 6.737 | 1 | .009 |
|  | Low Risk / Low Symptom | 0.113 | 1 | .737 |
| **Low Risk / Low Symptom**  (80) | High Risk / Low Symptom | 14.358 | 1 | < .001 |
|  | Low Risk / High Symptom | 9.679 | 1 | .002 |
|  | High Risk / High Symptom | 0.113 | 1 | .737 |

*Note.* Risk groups defined by median splits of SAVRY Total Score (High Risk vs. Low Risk) and BPRS Total Score (High Symptom vs. Low Symptom). P-values are derived from the Log Rank (Mantel-Cox) test.
